# Ecological correlation between diabetes hospitalizations and fine particulate matter in Italian provinces

**DOI:** 10.1186/s12889-015-2018-5

**Published:** 2015-07-25

**Authors:** Angelo G. Solimini, Maddalena D’Addario, Paolo Villari

**Affiliations:** Department of Public Health and Infectious Diseases, Sapienza University of Rome, Piazzale Aldo Moro 5, 00185 Rome, Italy

**Keywords:** Diabetes, PM2.5, Cross-sectional study, Hospitalization appropriateness

## Abstract

**Background:**

Exposure to particulate matter has been associated with increased risk of cardiovascular and respiratory diseases. We evaluated the ecological correlation between standardized hospital discharges with diabetes in Italian provinces and fine particulate matter (PM2.5) adjusting for common risk factors, socioeconomic factors and differences in hospitalization appropriateness.

**Methods:**

We used cross sectional data aggregated at the province level and available from official institutional databases for years 2008–2010. Covariates included prevalence of adult overweight, obese, smokers, physically inactive, education and income (as average gross domestic product per person, GDP). We reduced the number of covariates to a smaller number of factors for the subsequent statistical model by extracting meaningful components using principal component analysis (PCA). Log-linear multiple regression analysis was used to model diabetes hospital discharges with PCA components and PM2.5 levels and hospitalization appropriateness for men and women.

**Results:**

The first PCA components for both men and women were characterized by larger loadings of risk factors (obesity, overweight, physical inactivity, cigarette smoking) and lower socioeconomic factors (educational level and mean GDP). Diabetes hospitalization increases with the first PCA component and decreases with the index of hospitalization appropriateness. In fully adjusted models, diabetes hospitalizations increase with increasing annual PM2.5 concentrations, with a rise of 3.5 % (1.3 %–5.6 %) for men and of 4.0 % (1.5 %-6.4 %) for women per unit of PM2.5 increase.

**Conclusions:**

We found a significant ecological relationship between sex and age standardised hospital discharge with diabetes as principle diagnosis and mean annual PM2.5 concentrations in Italian provinces, once that covariates have been accounted for. The relationship was robust to different means of estimating PM2.5 exposure. A large portion of the variance of diabetes hospitalizations was linked to differences of hospital care appropriateness between Italian regions and this variable should routinely be included in ecological analyses of hospitalizations.

## Background

Fine particulate matter air pollution (PM2.5) is associated with an increased risk for cardiovascular events and this evidence is well supported in the literature [1]. Several studies demonstrate that during periods of high ambient air pollution, diabetic patients have increased susceptibility for vascular reactivity and doubled rates of hospital admission for heart disease [2, 3]. It has been suggested that some biological mechanisms related to air pollution exposure and cardiovascular diseases may also be involved in the onset of type 2 diabetes [4]. A recent experimental study on mice exposed to PM2.5 for 10 months (equivalent to a human exposure period of ~ 40 years) shows that this exposure was sufficient to cause insulin resistance and increased the type 2 diabetes prevalence and susceptibility to cardiovascular diseases [5].

There are now at least few epidemiologic studies showing some association between air pollution and diabetes, including a recent meta-analysis [6]. An ecological study has found that diabetes prevalence among adults in USA was greater in areas with higher PM2.5 concentrations [7]. This association was strong and the increased risk of diabetes was present even among areas that are below the EPA legal limits for PM2.5 [7]. In a German perspective cohort study, women exposed to higher levels of traffic-related air pollution (NO_2_ and PM) developed type 2 diabetes at a higher rate than controls with a risk increase over 16 years of follow-up by 15 % per 1 interquartile range of traffic-related PM exposure [8]. Another cohort study conducted in the Denmark suggested that the risk for diabetes was weakly and positively associated with increasing mean levels of traffic-related air pollution at the residence [9]. In contrast, associations between diabetes and exposure to particulate matter in the year before diagnosis, were not found in the Nurses’ Health Study [10]. However, an association between incident diabetes and residential proximity to a road was statistically significant [10].

In Italy there are well established evidences of the effect of increased air pollution levels on mortality for cardiovascular and respiratory diseases [11, 12]. Exposures to PM10 were positively associated with cardiovascular hospital admissions in 9 Italian cities [13]. A large cohort study on the most populated city of Italy (Rome) showed that long term exposure to NO2 and PM2.5 was associated with ischemic heart diseases, cardiovascular diseases and lung cancer [14]. Despite diabetes prevalence and mortality have been linked to its common risk factors on the Italian territory [15], there are not yet evidences if diabetes is also correlated with levels of air pollution.

Here we present a cross-sectional study assessing the ecological association between diabetes hospitalizations in Italy and ambient PM2.5 concentration at the province level. The relationship is adjusted for census level covariates for common risk factors, socio economic factors and for appropriateness of hospital admission with diabetes. All analyses were carried out from datasets retrieved from official and freely available databases.

## Methods

### Particulate matter

Annual levels of PM2.5 of Italian cities for the years 2008–2010 were obtained from hourly time series measured at all monitoring stations belonging to the regional monitoring networks (http://www.brace.sinanet.apat.it/web/struttura.html accessed on 31/07/2013). The time period and the monitoring stations were chosen to match the hospital discharge data at the province level of aggregation (see below). Only quality checked and validated measures from monitoring stations were retained, discharging data series with large portion of missing values. Mean, median, 95 percentile and maximum annual levels of PM2.5 were calculated from each monitor hourly time series belonging to 48 provinces (including all larger Italian cities), where 59.9 % of total Italian population was resident between 2008 and 2010. In total we used data from 129 monitors of different type (18 % off all monitors measured rural background pollution, 3 % industrial background, 44 % urban/suburban background, 31 % urban/suburban traffic, 5 % urban/suburban industrial). For the other provinces no PM2.5 data were available from the period 2008–2010. For sensitivity analysis we used the estimated concentrations of PM2.5 obtained using the National Integrated Model for 2005 [16, 17]. Mean concentration for the 48 provinces was estimated using image analysis from the 2005 national map of PM2.5 obtained using the dispersion model. The model simulates the dispersion and the chemical transformation of principal atmospheric pollutants concentrations at 4 km × 4 km resolution.

### Diabetes hospital discharge data

The most recent statistics belonging to years 2008 to 2010 were obtained from the official national database of the Italian National Institute of Statistics containing all discharges for acute cares from public and private hospitals at province level of aggregation [18]. Sex and age standardized hospital discharge data (reported as cases per 10,000 standard Italian population) with diabetes as principal diagnosis (ICD IX code: 250.x) was extracted for provinces matching particulate matter data. Only discharges of patients with age >45ys were included in our analysis. Three years average was calculated to increase data precision and statistical strength, following a similar study [7].

### Covariates

Sex and age standardised covariates at regional levels were obtained from routine national survey for the years 2008–2010 of the Italian National Institute of Statistics [18] and included percent of adults overweight, obese, smokers and physically inactive. Socioeconomic covariates included average of Gross Domestic Product (GDP) per individual and education as percentage of population with junior high school diploma (8 years of education or more). To control for patterns in diabetes hospitalizations that could be explained by factors other than PM2.5 and measured covariates, we also entered a qualitative variable classifying Italian regions into macro-regions (North-East, North-West and Centre-South). Additionally, due to the different levels of appropriateness of hospitalizations in Italian regions [19], we included an indicator variable for appropriateness of diabetes hospitalizations. As others ([20]), we used the standardised average length of hospital stay for diabetes with complications. This variable is among those routinely used by the Health Ministry to monitor the regional quality of primary care services (higher the value, worst is efficiency of primary care services for diabetic impatiens) and was dicotomically coded as appropriateness above or below the national average (in 2010: hospital stay of 40.1 days per 100,000 inhabitants, [20]).

### Statistical analysis

Bivariate correlations were preliminarily calculated among covariates. As expected, most covariates were correlated among each other and the subsequent regression modeling exercise would have been heavily affected by multicollinearity of the data. Therefore, we reduced the number of covariates to a smaller number of factors by principal component analysis (PCA) while retaining as much as possible of the variation present in the data set. Varimax rotation was used after factor extraction and scores were saved for first 2 factors. Visual examination of plots of factor 1 vs factor 2 and analysis of factor loadings (e.g. correlation between the original variables and the factors) were used to interpret in terms of covariates the extracted PCA components.

Standardised hospital discharge for diabetes was than regressed against factor scores, the index of appropriateness and mean, median, 95 percentile and maximum PM2.5 levels using standard least squares. Model diagnostics were carried out with residuals analysis, including check for possible spatial autocorrelation (which was not present in any model). In sensitivity analysis robust standard errors were used to estimate confidence intervals using Eicker-Huber-White outer product sandwich estimator as provided by R package Sandwich [21]. All statistical analysis was carried out with R 2.3.

## Results

Descriptive statistics of standardised hospital discharge with diabetes as primary diagnosis, PM2.5 levels and covariates for the years 2008–2010 are shown in Table 1. The dataset covers 48 Italian provinces, with a population of 34,249,361 registered residents (59.9 % of total Italian population). Population over 65 years of age ranged between 16.3 % and 30.5 % for women and between 12.4 % and 23.9 % for men. The 2008–2010 PM2.5 average levels in Italian provinces ranged between 11 μg/m^3^ and 32 μg/m^3^ with a mean of 20.1 μg/m^3^. Diabetes hospital discharge (cases >45y per 10,000 standard Italian population) for women ranged between 4.6 and 66.9 with a mean of 16.2; the range for men was between 8.4 and 83.8 with a mean of 23.4 Prevalence of women smokers ranged between 12.3 % and 20.1 %, obese between 6.8 % and 11.9 %, overweight between 22.9 % and 36.8 %, physically inactive 15.5 % and 58.9 %, higher education 65.6 % and 78.0 %. Prevalence of men smokers ranged between 23.2 % and 32.7 %, obese between 6.4 % and 13.6 %, overweight between 40.9 % and 49.3 %, physically inactive between 13.7 % and 48.3 %, higher education 76.8 % and 87.1 %.

**Table 1:** Descriptive statistics of population, covariates, PM2.5 and diabetes hospital discharge from 48 Italian provinces in 2008-2010

	Minimum	Maximum	Mean	Std. Deviation
*Women residents*	64817.3	2157796.0	370685.0	435084.1
>65 years (%)	16.3	30.5	24.1	2.9
Smokers (%)	12.3	20.1	17.7	1.9
Obese (%)	6.8	11.9	9.1	1.4
Overweight (%)	22.9	36.8	27.3	3.1
Physically inactive (%)	15.6	58.9	38.7	9.4
At least junior high school diploma (%)	65.6	78.0	71.7	3.3
Hospital discharge with diabetes as primary diagnosis (cases >45ys per 10,000 standard Italian population)	8.4	83.9	23.6	13.4
*Men residents*	62528.3	1973046.0	348823.1	403887.9
>65 years (%)	12.4	23.9	18.5	2.4
Smokers (%)	23.2	32.7	28.2	2.0
Obese (%)	6.4	13.6	11.3	1.6
Overweight (%)	40.9	49.3	43.9	2.4
Physically inactive (%)	13.7	48.3	30.5	7.6
At least junior high school diploma (%)	76.8	87.1	80.8	2.6
Hospital discharge with diabetes as primary diagnosis (cases >45ys per 10,000 standard Italian population)	4.6	66.9	16.4	11.3
Average GDP per individual (Euro)	12776.4	29065.8	23988.0	4507.5
Average length of inappropriate hospital stay for diabetes (days per 10,000 standard Italian population)	0.0	77.4	32.2	17.5
Number of PM2.5 monitors per province	1	10	2.5	2.0
Average annual PM2.5 (ug/m3)	11.0	32.0	20.1	5.3

The PCA factor loadings (e.g. correlation between each covariate and the rotated component) of the first components are reported in Table 2. In the men PCA, the first 2 principal components explained 76.1 % of total cumulative variance while in the women PCA explained 75.6 % of total cumulative variance and all other components added little explained variance and were negligible. Increasing values of PC1 are interpretable as larger proportion of population with risk factors and lower education and income (Table 2).

**Table 2:** Factor loadings of covariates on PCA components (PC1 and PC2)

Gender	Variable	Load on PC1	Load on PC2
Men	% Smokers	0.84	−0.01
	% Physically inactive	0.95	0.08
	% Obese	0.03	0.83
	% Overweight	0.83	0.33
	% High education	−0.20	−0.74
	Mean individual GDP	−0.91	−0.24
Women	% Smokers	−0.12	0.94
	% Physically inactive	0.67	−0.56
	% Obese	0.65	−0.36
	% Overweight	0.90	−0.03
	% High education	−0.77	0.17
	Mean individual GDP	−0.79	0.53

The regression models are summarized in Table 3. The men model explained 43 % of total variance and was highly significant (p < 0.001). Macro-region (*p* = 0.3) and PC2 (*p* = 0.9) were not significant and were dropped from the final model. The log of hospital discharge for diabetes in Italian provinces increases with PC1 (b = 0.170; 95 % CI = 0.042; 0.297), PM2.5 (b = 0.035; 95 % CI = 0.013; 0.056) and decreases with hospitalization appropriateness (b = −0.428; 95 % CI = −0.733; −0.123). The women model explained 47 % of total variance and was highly significant (p < 0.001). Macro-region (*p* = 0.1) and PC2 (*p* = 0.2) were not significant and were dropped from the final model. The log of hospital discharge with diabetes in Italian provinces increases with PC1 (b = 0.149; 95 % CI = 0.015; 0.289), PM2.5 (b = 0.040; 95 % CI = 0.015; 0.064) and decreases with hospitalization appropriateness (b = −0.728; 95 % CI = −1.039; −0.417).

**Table 3:** Log-linear regression models of hospital discharge with diabetes as primary diagnosis, covariates and mean annual PM2.5 in Italian provinces

Gender	Variable	b (95 % CI)	P value
Men	Intercept	2.664 (2.223,3.104)	<0.001
	PCA Factor 1	0.170 (0.042,0.307)	0.009
	Appropriate hospitalization (=yes)	−0.428 (−0.733,-0.123)	0.006
	Annual PM2.5	0.035 (0.013-0.056)	0.002
Women	Intercept	2.382 (1.882-2.882)	<0.001
	PCA Factor 1	0.149 (0.015-0.289)	0.029
	Appropriate hospitalization (=yes)	−0.728 (−1.039, −0.417)	<0.001
	Annual PM2.5	0.040 (0.015-0.064)	0.001

Model R^2^ were 0.43 for men and 0.47 for women

As sensitivity analysis, we repeated the modeling exercise using different estimates of PM2.5 exposure in the fully adjusted models and providing robust confidence intervals obtained from “sandwich” estimates of SE (Table 4). The PM2.5 term remained significant in both, men and women models, when using the median, the 95 percentile or the annual mean estimated from the 4 × 4 km (Table 4).

**Table 4:** Sensitivity analysis of Log-linear regression models of hospital discharge with diabetes as primary diagnosis and PM2.5 in Italian provinces

Gender	PM2.5 estimate	b (95 % CI)	Sandwich 95 % CI
Men	Annual mean	0.035 (0.013, 0.056)	(0.012, 0.058)
	Annual median	0.053 (0.033, 0.083)	(0.026, 0.080)
	Annual 95 percentile	0.011 (0.003, 0.019)	(0.001, 0.020)
	Annual mean estimated from model	0.003 (0.001, 0.005)	(0.001, 0.005)
Women	Annual mean	0.040 (0.015, 0.064)	(0.012, 0.058)
	Annual median	0.064 (0.031, 0.097)	(0.032, 0.096)
	Annual 95 percentile	0.012 (0.003, 0.021)	(0.002, 0.022)
	Annual mean estimated from model	0.003 (0.001, 0.006)	(0.001, 0.006)

All models adjusted for PC1 and appropriateness of diabetes hospitalization. Robust standard errors were used to estimate sandwich 95 % confidence intervals [21]

## Discussion and conclusions

Type 2 diabetes mellitus has been shown to be linked with some risk factors like gender [22], ageing [23], overweight or obesity [24], physical inactivity [25], ethnicity [26], genetic factors [27] and others [28]. More recently, several studies have also suggested a potential role of particulate matter in air pollution, in the development of diabetes. Biological plausible mechanisms include, among others, increased systemic oxydative stress and inflammatory responses to air pollutants (reviewed elsewhere [29, 30]). In animal models, exposure to PM2.5 resulted in adipose inflammatory changes (pro-inflammatory to anti-inflammatory macrophage ratio and insulin signalling abnormalities) which could lead to insulin resistance [5, 31]. Recent research identified cellular mechanisms that are implied in insulin resistance development caused by joint exposure to high fat diet and high levels of PM2.5 [32]. Human studies have given mixed evidences of the association between particulate matter and systemic inflammation and/or insulin resistance possibly due to differences in study populations, exposure levels and specific markers examined [2, 33–35].

In our analysis we used PCA to reduce the (correlated) variables describing the various risk and socioeconomic factors into a synthetic variable (PC1) for subsequent modelling with linear regression. Obesity, overweight, physical inactivity, cigarette smoking were all positively correlated with PC1 while educational level and GDP were inversely related. As expected, diabetes hospital discharge was positively related with PC1 and inversely related to hospitalization appropriateness in both male and female models. Regarding particulate matter pollution, our ecological analysis showed that diabetes hospitalizations (of 45 years or older age) for men increase by 3.5 % and women increase by approximately 4 % each unit increase of PM2.5.

Epidemiological studies examining the association between air pollution from traffic emissions and incidence or prevalence of diabetes are scarce and produced non consistent results. In the Toronto area, [4] used hospital discharges to estimate prevalence and found only for women an increased prevalence by 17 % across the interquartile range of NO2. Contrary to those findings, a cross-sectional ecological study conducted in the Netherlands [36] found no associations between diabetes prevalence (assessed through self reported previous diagnosis of the diabetic condition by a doctor) and exposure to traffic related pollution (though there were some indications for a relation with traffic in a 250 m buffer from the residential address of participants). A more recent study in the US found a 1 % diabetes prevalence (estimated from self reporting of diabetes diagnosis by participants) with an increase by 10 μg/m^3^ of PM2.5, after adjusting for many risk factors [7]. By comparison, our estimated increase per 10 μg/m^3^ PM2.5 is much higher (35 % for men and 40 % for women). However, the PM2.5 concentration range examined in [7] is lower, having a range between 2.5–17.7 μg/m^3^ (with median = 11 μg/m^3^) while in our study we had a larger range of PM2.5 (11–32 μg/m^3^) and a higher median value (18.68 μg/m^3^) that may explain the difference in diabetes increase. Furthermore, diabetes prevalence in [7] is based on self reported previous diagnosis of the diabetic condition by a doctor collected through a population survey, while we based our study on hospital discharge data. Prevalence estimates based on self reporting from individuals such as in surveys may miss those individuals that are yet not aware of the disease. Also hospital discharge data with diabetes as principal diagnosis possibly underestimate the true population prevalence due to the comorbidity of diabetes with cardiovascular conditions that are often the primary diagnosis of hospitalization. It is also true that many general practitioners nowadays screen for insulin resistance those (especially if of older age) individuals carrying some risk factors, therefore increasing their likelihood of hospitalization.

To date we are aware of 5 cohort studies that examined the association between air pollution and incidence of type 2 diabetes. [8] found a significant increase of the hazard for incident diabetes of 15–42 % per interquartile range of traffic-related exposure (PM10, NO2) in a women cohort from the highly industrialized Ruhr district in Western Germany. More recently, in Ontario (Canada), [37] found an 11 % increased diabetes incidence rate by a 10 μg/m3 increased PM2.5 level in a cohort of more than 60,000 participants. Contrary to those findings, [38] did not found significant association between incident diabetes and PM2.5, while they found an increase by 25 % of incident diabetes with interquartile range increase of NO2 (12.4 ppb) in a cohort of black women living in Los Angeles. No associations of incident diabetes with PM2.5 or PM10 were found in the analysis of 2 prospective cohorts in the US [10], although an association with distance to road (used as proxy for exposure to traffic related pollution) was reported. Finally another recent cohort study conducted in Denmark found a borderline statistical association between confirmed cases of diabetes and NO2 levels [9]. Notably, the risk was higher between non smokers and physically active people.

In Italy, hospital use by diabetic patients is substantially greater than that by the non-diabetic population [39]. The persistence of sub-optimal care can be responsible for an increased risk of complications, which in turn determines an increased demand for hospital care [40]. Therefore, excess hospitalization and longer hospital stays for patients with diabetic complications can be seen as an indicator of the quality of primary diabetes care [41]. In our ecological analysis the indicator variable of hospital length of stay for diabetes with complications (routinely used as appropriateness index by the Ministry of Health to monitor primary health care) was highly significant. Therefore, we advocate that an index of appropriate hospitalization should always be considered in ecological analyses dealing with hospital discharges. Previous analysis of hospital admissions for diabetes in Italy in different years has shown a decreasing trend from 2005 (13.7 per1000 inhabitants) to 2009 (12.0 per 1000 inhabitants). While this effect may be connected with increased appropriate hospitalization of diabetic patients and better management of primary cares, still Italian regions exhibit large differences in hospital discharge [19].

Ecological analyses are affected by well known limitations and our study does not allow us to conclude about causal relationship between higher fine particulate matter levels and increased hospital discharge diabetes in the Italian territory. It is important to remember that linear relationship at ecological aggregated level could not hold at the individual level and/or might be non linear. Our analysis was based on official and validated data and covers roughly 2 thirds of the Italian population and all major urban areas. Additionally, we used a cross sectional approach by adopting a 3 year averaging of all variables to increase the statistical strength, as suggested in a similar study [7]. We used covariates available from official surveys designed for providing meaningful data at the regional level of aggregation therefore assuming that the average characteristics are representative of the population in that area. Furthermore, we assumed that average exposure of individuals can be estimated from PM2.5 levels from all ground monitoring stations on a given territory. In sensitivity analysis we entered in models different metrics for annual PM2.5 measured levels (mean, median, 95 percentile and maximum) and levels estimated from a dynamic model with 4 × 4 km of resolution and still resulted significant terms. Like others [7], we also note that it is possible that the hospitals attracting more diabetic patients are placed in most polluted areas. However, when we entered in the models a term correcting for geographical macro-region (North East, North West, South) aiming in capturing residual unexplained geographical variation in hospital discharges, still the PM2.5 term remained significant. Finally, we could have omitted variables that could explain part of the observed relationship between PM2.5 and diabetes hospital discharges such as NO2 concentrations that are reported to have the same ecological relationship with diabetes prevalence.

In conclusion, we have found a significant ecological relationship between sex and age standardised hospital discharge with diabetes as principle diagnosis and PM2.5 concentrations in Italian provinces, once that covariates have been accounted for. The relationship was robust to different means of estimating PM2.5 exposure. A large portion of the variance of diabetes hospitalizations was linked to differences of primary care appropriateness between Italian regions and this variable should routinely be included in ecological analyses of hospitalizations.
